# Functionalization of Bacterial Microcompartment Shell Proteins With Covalently Attached Heme

**DOI:** 10.3389/fbioe.2019.00432

**Published:** 2020-01-09

**Authors:** Jingcheng Huang, Bryan H. Ferlez, Eric J. Young, Cheryl A. Kerfeld, David M. Kramer, Daniel C. Ducat

**Affiliations:** ^1^MSU-DOE Plant Research Laboratory, Michigan State University, East Lansing, MI, United States; ^2^Department of Biochemistry & Molecular Biology, Michigan State University, East Lansing, MI, United States; ^3^Environmental Genomics and Systems Biology and Molecular Biophysics and Integrated Bioimaging Divisions, Lawrence Berkeley National Laboratory, Berkeley, CA, United States

**Keywords:** heme functionalization, cytochrome, self-assembly, protein engineering, synthetic biology, electron transfer

## Abstract

Heme is a versatile redox cofactor that has considerable potential for synthetic biology and bioelectronic applications. The capacity to functionalize non-heme-binding proteins with covalently bound heme moieties *in vivo* could expand the variety of bioelectronic materials, particularly if hemes could be attached at defined locations so as to facilitate position-sensitive processes like electron transfer. In this study, we utilized the cytochrome maturation system I to develop a simple approach that enables incorporation of hemes into the backbone of target proteins *in vivo*. We tested our methodology by targeting the self-assembling bacterial microcompartment shell proteins, and inserting functional hemes at multiple locations in the protein backbone. We found substitution of three amino acids on the target proteins promoted heme attachment with high occupancy. Spectroscopic measurements suggested these modified proteins covalently bind low-spin hemes, with relative low redox midpoint potentials (about −210 mV vs. SHE). Heme-modified shell proteins partially retained their self-assembly properties, including the capacity to hexamerize, and form inter-hexamer attachments. Heme-bound shell proteins demonstrated the capacity to integrate into higher-order shell assemblies, however, the structural features of these macromolecular complexes was sometimes altered. Altogether, we report a versatile strategy for generating electron-conductive cytochromes from structurally-defined proteins, and provide design considerations on how heme incorporation may interface with native assembly properties in engineered proteins.

## Introduction

Efficient electron transport through protein redox carriers is required for many energy-converting biological processes, and achieving this requires the close positioning of donor-acceptor pairs and fine control of redox potential, as exemplified by the electron transport chain in photosynthetic reaction center (Blankenship, 2014). Changes in position of even a few Angstroms can change the rates of electron transfer by orders of magnitude, dramatically influencing the rates and yield of native biological redox reactions (Rutherford et al., 2012).

It is also critical to consider these factors when designing redox proteins for the growing field of bioelectronics (Page et al., 1999), where biological molecules are used as components (e.g., bionanowires, biocapacitors, light capture or biosensors) in novel electronic devices. One design principle for producing a long-range electron transport device, inspired by the natural microbial nanowire (Reardon and Mueller, 2013), is to use a self-assembling protein scaffold to spatially organize redox domains into close proximity and with suitable orientation relative to one another. For example, the redox biofilm reported by Altamura et al. utilized a fusion protein containing elements of a rubredoxin (redox domain) and prion (self-assembling domain) to produce a redox active biofilm (Altamura et al., 2017). Yet, this system required a flexible linker to connect two protein domains, and such unstructured domains can cause unwanted mobility between the tethered redox centers (Altamura et al., 2017). Flexible (or undefined) positioning of redox carriers can create additional layers of complexity in the analysis and optimization of long-range electron transport in bioelectronic materials. Therefore, strategies that allow positioning of redox co-factors in structurally defined locations of a biomaterial are ideal.

**Figure 1:**
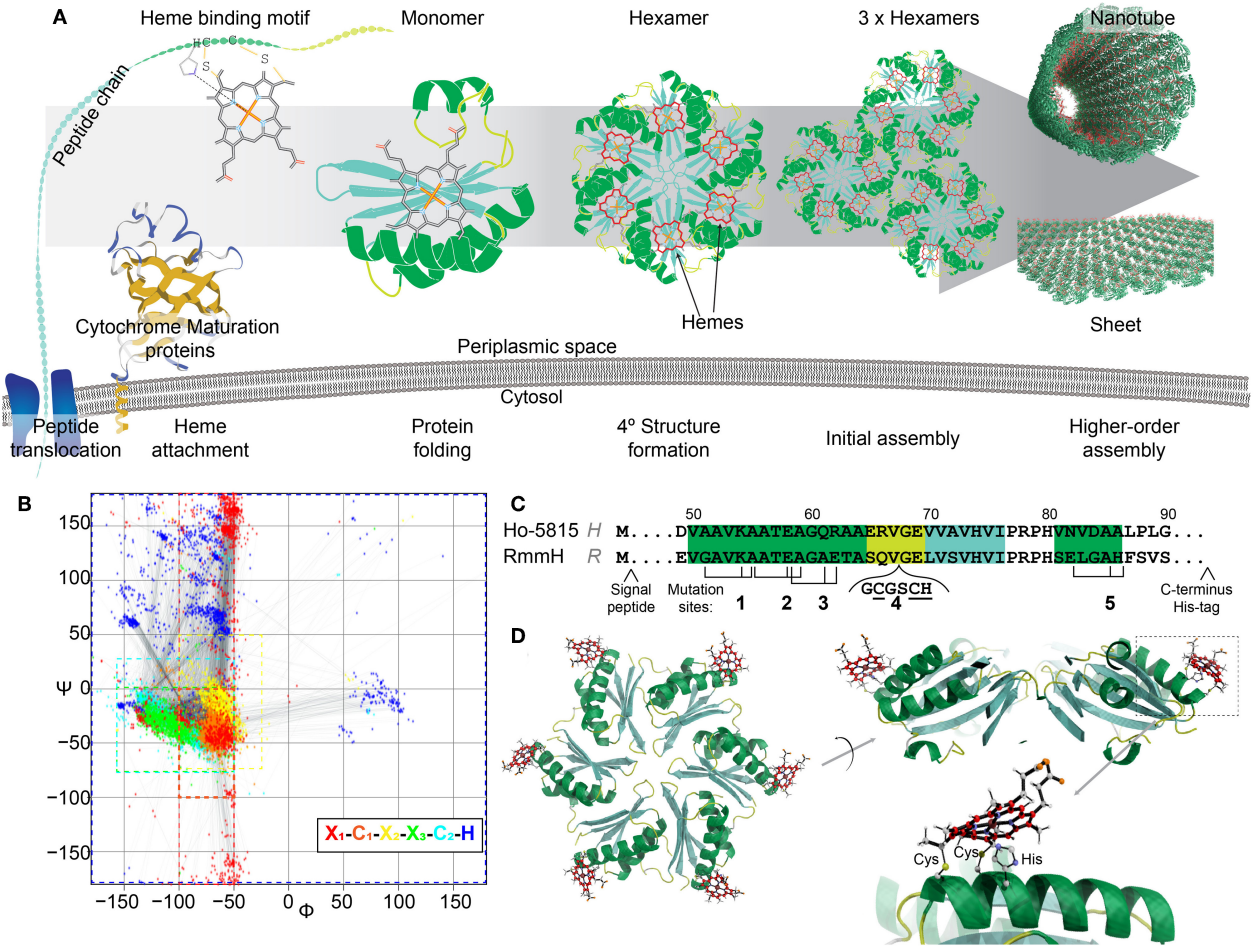
Design of heme-functionalized BMC-H shell proteins. **(A)** Cartoon schematic showing a hypothetical pathway of a heme from attachment on a shell protein to the formation of large structures. **(B)** Ramachandran plot of torsion angles within the heme binding motifs of naturally occurring cytochrome *c* structures (*n* = 249 heme binding motifs). Each color represents one amino acid in the motif. **(C)** Partial protein sequences of Ho-5815 and RmmH shell proteins. Secondary structures are highlighted (green: helix, yellow: loop, cyan: sheet). Three amino acid residues (black ticks) at Mutation sites 1,2,3, and 5 were changed to generate a heme binding motif (CxxCH). Six amino acids were inserted at mutation site 4 to introduce the heme binding motif at the loop region. **(D)** Molecular dynamics model of BMC-H variant **R**_1_ (heme at Mutation site 1 in RmmH). Conjugated carbon atoms in hemes are shown in red.

The goal of the present study was to develop a rationally-designed model system for electron transport over longer distances by attaching heme at targeted, spatially-defined positions within the existing backbone of self-assembling proteins *in vivo*. Heme is a common biological redox cofactor important for a diverse array of endogenous electron transfer reactions (Chapman et al., 1997). *c*-type heme, found in cytochrome *c*, has two side chains (position 2 and 4) that form thioether bonds with two cysteine residues within the heme binding motif (mostly CxxCH, where C is cysteine, H is histidine and x can be any residue; Figure 1A). The histidine in the motif acts as one of two axial ligands to the iron center of the heme. This covalent linkage is believed to influence protein stability, tune redox potentials, and support conformations that allow hemes to be surface-exposed (Bowman and Bren, 2008).

The covalent attachment of heme to the binding motif is catalyzed by a dedicated system called the cytochrome *c* maturation (Ccm) system (Kranz et al., 2009). Ccm system I (Ccm-I) is found in gram-negative bacteria (Thony-Meyer et al., 1995), has the broadest protein-substrate diversity, and can recognize the heme binding motif within a large variety of *apo*-cytochromes (Allen and Ferguson, 2006), making it a promising posttranslational modification tool for protein engineering. Ccm-I requires the Sec protein secretion pathway to translocate nascent polypeptide chains through the plasma membrane into the periplasmic space before a heme will be attached (Kranz et al., 2009). It has been hypothesized that Ccm-I will attempt to attach a heme to any CxxCH motif of any protein that is delivered by the Sec pathway, independently of any other context within the polypeptide chain (Allen and Ferguson, 2006). Thus, it would be feasible to attach a functional heme to any target protein that is secreted by mutating three amino acids into the CxxCH attachment motif, assuming that the residue substitutions and attachment of the heme permit the protein to fold properly. This approach would potentially allow redox features to be appended onto proteins at defined locations (Figure 1A), and could circumvent the sophisticated process of *de novo* design of heme binding pockets.

In this work, we utilized the Ccm-I to attach hemes onto the self-assembling shell proteins of a bacterial microcompartment (BMC). BMCs are proteinaceous bacterial organelles that encapsulate enzymes that catalyze specialized reactions within a protein shell (Cheng et al., 2008; Axen et al., 2014; Kerfeld et al., 2018). Aside from enclosed compartments, these shell proteins can also self-assemble into other super-molecular structures naturally (e.g., sheets, stripes, or tubes; Figure 1A), many of which could be ideal scaffolds for other biological and nanotechnology applications (Lassila et al., 2014; Noël et al., 2016; Young et al., 2017; Lee et al., 2018; Plegaria and Kerfeld, 2018; Planamente and Frank, 2019). Toward the construction of heme-based synthetic bionanowires, we chose two shell proteins known to form nanotubes *in vivo* and *in vitro* as our protein engineering targets. We demonstrate the attachment of one or two hemes directly onto the shell protein subunits, and present evidence that appending these redox cofactors does not eliminate their self-assembly capabilities.

## Results

### Design of Heme Attachment Sites on Shell Proteins

Among the BMC shell proteins, one protein family is known to form homo-hexamers (BMC-H) that natively self-assemble into large structures (e.g., the facets of endogenous BMCs) (Kerfeld et al., 2005, 2018; Axen et al., 2014; Sutter et al., 2017). Many reports have shown that when BMC-H proteins are expressed individually outside of their native context, they can form alternative higher-order protein assemblies, such as tubes or sheets (Lassila et al., 2014; Noël et al., 2016; Sutter et al., 2016; Young et al., 2017; Uddin et al., 2018). However, it was also found that these higher-order assemblies are sensitive to minor amino acid changes within individual shell proteins, especially to those residues that mediate lateral hexamer-hexamer interactions (Pang et al., 2014; Sinha et al., 2014; Young et al., 2017). For example, the wild-type (WT) BMC-H protein from *Haliangium ochraceum* (Ho-5815, HO BMC-H) forms sheets *in vitro* (Lassila et al., 2014; Sutter et al., 2016), but the Y41A mutant forms nanotubes (Supplementary Figure 1). We selected two nanotube-forming BMC-H proteins as candidates for appending heme cofactors; one of these proteins, MSM_0272 (also known as RmmH, *Rhodococcus* and *Mycobacterium* microcompartment BMC-H) has been previously shown to robustly form nanotubes (Noël et al., 2016; Mallette and Kimber, 2017), and the second shell protein is a Y41A mutant of Ho-5815 that we found to form nanotubes when expressed in *Escherichia coli* (*E. coli*) (Supplementary Figure 1).

To determine potential heme attachment sites, we first searched the preferred secondary structure of heme binding motifs within natural cytochromes deposited in Protein Databank (PDB) to gain insight into structural features that naturally support a ligated heme. The attachment of a rigid heme to the CxxCH motif on an unfolded protein likely constrains the possible secondary structures that can be supported (Negron et al., 2009). Therefore, we analyzed the torsion angles of the residues in the heme binding motif (x_1_C_1_x_2_x_3_C_2_H, where x_1_ is the residue before the heme binding motif, and the x_2_, x_3_ are the first and the second residue between two C) of 180 *c*-type cytochromes within the PDB, which can be visualized as a Ramachandran plot (Figure 1B, Supplementary Table 1). In native *c*-type cytochromes, the torsion angles of residues C_1_x_2_x_3_C_2_ in the motif are closely matched to those in an α-helical structure (−50° < ψ < 0°, φ < −50°), although the torsion angles in x_1_ or H have more variability (Figure 1B; red or dark blue, respectively). Based on this information about torsion angles in natural cytochromes, we searched for similar secondary structures within the conserved pfam00936 domain of BMC-H proteins. After checking for potential intra-/inter-protein clashes after heme attachment, we identified four mutation sites that are compatible with the introduction of heme binding motifs (Figure 1C). At each mutation site, up to three point-mutations were introduced to create the CxxCH motif, while the residues between the cysteines remained unmodified. As an additional attachment site, a loop region (site 4) (Jorda et al., 2016) was chosen for inserting the heme binding motif. Molecular dynamics simulation (Supplementary Figure 2A) of the predicted motion of hemes attached at these 5 sites (i.e., the RMSD of the central iron atom) was comparable to the flexibility of atoms within the protein backbone itself (~1–2 Å in 800 ps of simulated time; Supplementary Figure 2B), suggesting these sites offer a relatively structurally-defined location for heme attachment.

We performed site-directed mutagenesis to insert the heme attachment motif on two BMC-H candidates: the Y41A mutant of Ho-5815 (**H**-variants) and the WT RmmH (**R**-variants) (Figure 1C). Based on crystal structures of the wild-type shell proteins (Sutter et al., 2016), sites 1,2,3, and 4 are located at the convex side of the hexamer (Figure 1D), while a heme coordinated at site 5 is predicted to face the concave side (Supplementary Figure 2A). An N-terminal periplasmic targeting signal peptide from small tetraheme cytochrome (Leys et al., 2002) (STC) in *Shewanella oneidensis* MR-1 (SO_2727) and a C-terminal 6xHis-tag were also appended to complete each expression construct (Supplementary Table 2). Each construct was expressed in *E. coli* BL21 with Ccm-I gene cassette encoded by the pEC86 plasmid (Arslan et al., 1998).

### Covalent Heme Attachment to Shell Proteins

**Figure 2:**
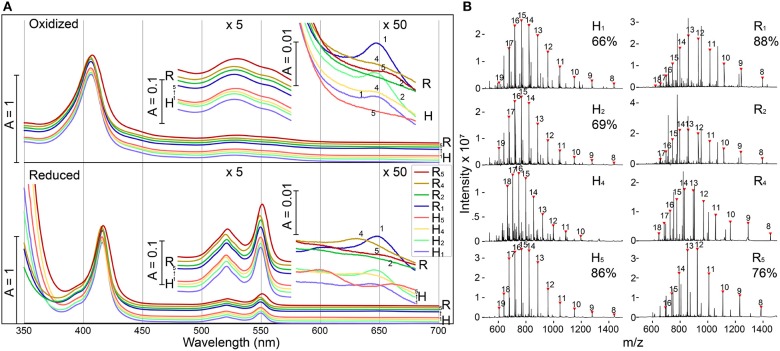
Modified shell proteins contain redox-active hemes **(A)** UV-Vis spectra of purified shell-cytochromes under oxidizing (upper) or reducing (lower) conditions. Green region and red region also plotted with 5x or 50x amplification. **(B)** Protein mass spectrometry of purified shell-cytochromes. Expected *holo*-protein are marked with charge states in red triangles. The percentage of *holo*-protein in all full-length protein are labeled under the sample name (If available, see section Methods). See Supplementary Figure 3 for unlabeled peaks.

*E. coli* expressing four of the five modified shell proteins of both the **H**- and **R**- variants (mutation sites 1,2,4, and 5) exhibited vivid red pigmentation of the cell pellets, indicating likely heme attachment. Attempts to express shell proteins modified at site 3 for both **H**- and **R**- variants did not yield a pellet with red pigmentation, and thus these constructs were excluded from further investigation. Affinity chromatography was used to isolate each of the eight recombinant proteins, and the purified fractions exhibited characteristic spectral features of a covalently-bound, low-spin, *c*-type heme in a ferric state (Butt and Keilin, 1962), including an intense Soret band at 407 nm as well as a broad Q-band peaking at 530 nm (Figure 2A). Reducing the purified protein with sodium dithionite, induced a red-shift in the Soret peak to 416 nm and the emergence of two Q-bands at 521 nm and 550 nm, as expected for low-spin heme. In the red region, **H**_1_, **H**_2_, and **R**_1_ (Each subscript indicates one heme binding motif at the corresponding mutation site in Figure 1C) each possesses a small absorbance band around 650 nm, suggesting the presence of a small amount of high-spin heme with weak-field ligands (e.g., water), while this band is absent in other samples, indicating **H**_4_, **H**_5_, **R**_2_, **R**_4_, and **R**_5_ contain mainly low-spin hemes with strong-field ligands. Although the functional group that could serve as the sixth heme ligand was not certain, protein residues (e.g., His, Lys) or imidazole in the purification buffer could potentially serve as the strong-field axial ligand. Altogether, these spectral features indicate the presence of *c*-type hemes (Butt and Keilin, 1962) in these modified shell proteins (Hereafter, we refer to these recombinant proteins as “shell-cytochromes”).

The identity of shell-cytochromes and the position of covalently attached hemes were further confirmed by intact protein mass spectrometry (MS) under denaturing conditions (Figure 2B). In all eight purified shell-cytochromes, the expected molecular weights of monomeric *holo*-proteins were observed (Figure 2B). Additionally, MS revealed that some purified shell-cytochrome samples contained a relatively minor fraction of *apo*-proteins without attached heme (Supplementary Figure 3). In **H**_1_, **H**_2_, **H**_5_, **R**_1_, and **R**_5_, the relative amount of *apo*-protein was measurable and the percentages of the *holo*-protein in the total full-length shell proteins were 66%, 69%, 86%, 88%, and 76% respectively. In **H**_4_, **R**_2_, and **R**_4_ shell-cytochrome samples, a minor amount of *apo*-protein was detectable, but it could not be quantified accurately by MS (likely >90% heme occupancy).

The redox midpoint potentials of two representative shell-cytochromes **R**_1_ and **R**_5_ were determined by equilibrium redox titrations followed by observation of absorbance changes at ~550 nm, which is indicative of the redox state of *c*-type hemes (Leslie Dutton, 1978). Fitting the titration curve data with a one-component single-electron (*n* = 1) Nernst equation yielded a ${\text{E}}_{\text{m}}^{\prime }$ (vs. SHE) of −215 ± 2 mV and −219 ± 3 mV, for **R**_1_ and **R**_5_ respectively (Supplementary Figure 4). These ${\text{E}}_{\text{m}}^{\prime }$ values are close to the model heme peptide Microperoxidase with bis-imidazole ligand (−206 mV; Zamponi et al., 1990), suggesting a fully solvent exposed *c*-type heme with two imidazole (or similar) ligands. However, the titration curves appeared slightly broader than what could be explained with a single (*n* = 1) redox component fitting curve (Supplementary Figures 4B,D), potentially indicating the hemes were in heterogeneous microenvironments (Battistuzzi et al., 2002), possibly because of alternative ligands or protein folding conformations. Nevertheless, the relative negative redox midpoint potentials of the tested shell-cytochromes suggested that common electron donor like NAD(P)H or ferredoxin could donate electrons to the hemes, and electrons in the hemes should have comparable energy to drive bioindustrially-relevant processes, such as the reduction of acetaldehyde to ethanol (Loach, 1976).

To increase heme density, shell protein variants with two heme binding motifs in each monomer were constructed (**H**_2+5_, **H**_2+4_, **H**_4+5_, **R**_2+5_, **R**_2+4_, and **R**_4+5_). Upon expression, three (**H**_2+5_, **R**_2+5_, and **R**_4+5_) variants yield vivid red cell pellets and the shell-cytochromes were purified from these samples for further analysis. UV-Vis spectra suggested these diheme shell-cytochromes also possess *c*-type low-spin hemes, with comparable features to the shell-cytochromes ligated with a single heme (Supplementary Figure 5).

### Shell-Cytochromes Form Hexamer-Like Oligomers

**Figure 3:**
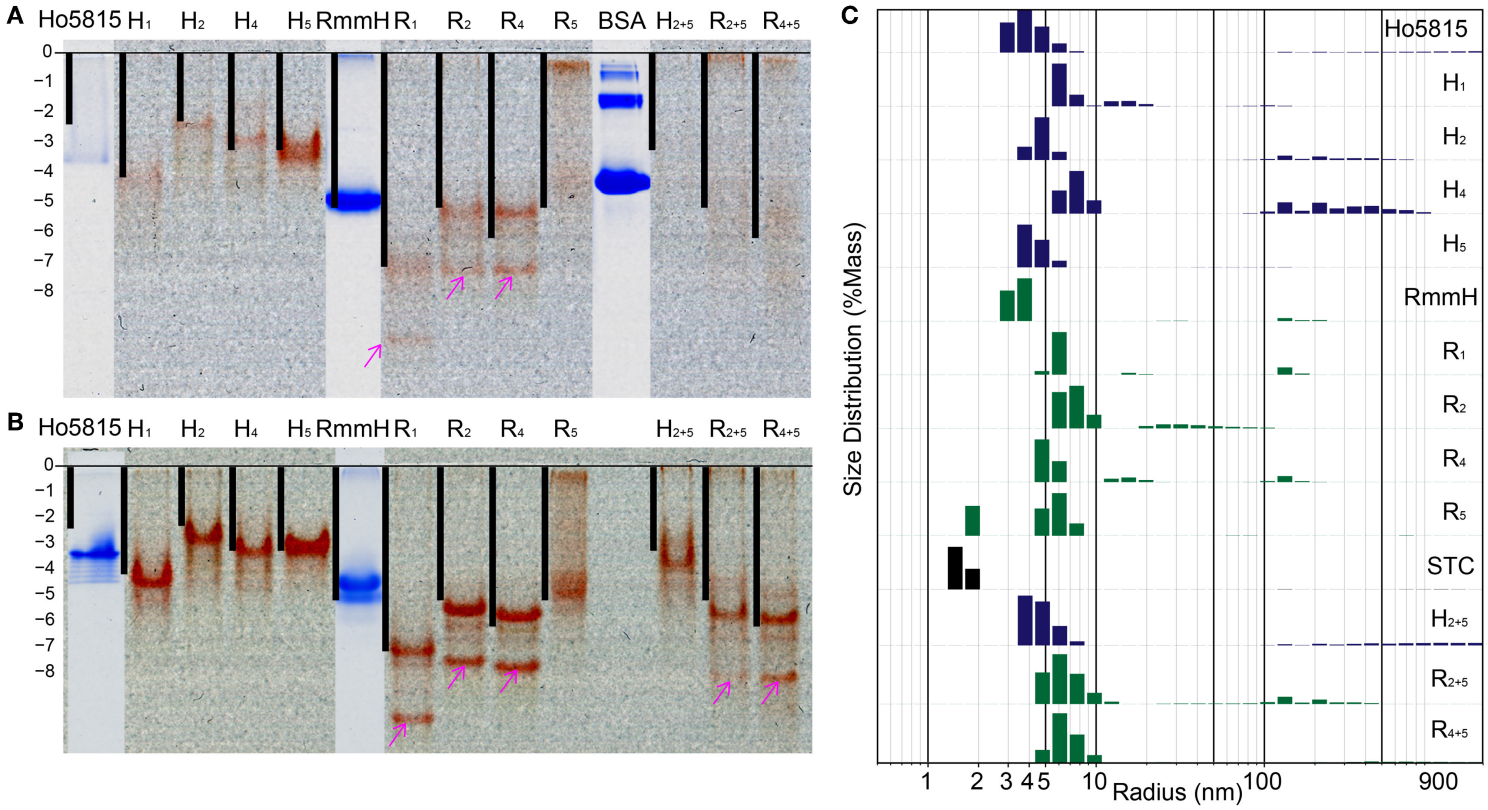
Shell-cytochrome retain capacity to oligomerize. **(A)** Native PAGE **(B)** native PAGE with 4 M urea show the mobility of **H**-/**R**-variants and their corresponding WT. Lanes of **H**-/**R**-variants show as un-stained and the red is the natural color of hemes. WT Ho-5815 and RmmH as well as molecule weight marker BSA were overlapped from the same gel after staining. The calculated net charge for shell-cytochrome monomers are plotted as black bars. The “0” charge is aligned to the beginning of resolving gel and the vertical length is stretched to allow RmmH to align to the gel band. **(C)** DLS of **H**-/**R**-variants compare with corresponding WT and STC. The heights of bars represent the relative mass distribution.

BMC-H proteins typically form stable hexamers with 6-fold rotational symmetry (Figure 1A), and the formation of these hexagonal tiles is the first step before self-assembly into higher-order structures (Kerfeld et al., 2005, 2018; Sutter et al., 2016, 2017; Young et al., 2017; Greber et al., 2019). We next examined whether shell-cytochromes retain the capacity to oligomerize, as do the WT shell proteins. On native polyacrylamide gel electrophoresis (PAGE), the heme-attached variants have similar, or slightly higher, mobilities in comparison to their WT (i.e., unmodified) variant (Figure 3A, Supplementary Figure 6A). After calculating the theoretical net charges for all samples, we found the mobilities of the major bands of shell-cytochrome samples followed the trend of net charges, suggesting that changes in the protein migration on native PAGE can be explained solely by the changes in charge introduced by incorporating the CxxCH heme motif (Figure 3A and Supplementary Figure 6A). This suggested that the shell-cytochromes form structures with similar size to the hexameric WTs.

We noticed that most of the shell-cytochromes (except **H**_5_) did not yield sharp bands on native PAGE (Figure 3A), suggesting the presence of hexamer-hexamer interactions. To get a clear separation of the stable oligomeric state of the shell-cytochromes, we supplemented 4 M urea to the gels to disrupt the potential weak hexamer-hexamer associations, and we found sharp bands with a similar net charge-dependent migration pattern (Figure 3B, Supplementary Figure 6B). Other than the expected red bands, we observed several secondary bands on the native PAGE, including a fast-moving heme-containing product (Figures 3A,B, Supplementary Figures 6A,B pink arrows), which MS analysis confirmed to be a degradation product lacking the first 27 amino acids (Supplementary Figure 3, Supplementary Table 2). Finally, a band that did not contain heme was observed, consistent with the fraction of *apo*-proteins also identified by MS (Supplementary Figures 6A,B cyan arrows, Supplementary Table 2).

We utilized dynamic light scattering (DLS) for further evaluation of the particle size distribution of shell-cytochrome samples. All the shell-cytochromes have similar (e.g., **H**_5_) or slightly larger average particle size compared to the WT hexamer, which has a radius of 3.5 nm based on X-ray crystallography structures (Mallette and Kimber, 2017) (Figure 3C). By contrast, the radius of STC, which has a similar molecular weight (12,122 Da) to a BMC-H monomer, but does not oligomerize, was measured to be <2 nm by DLS (Figure 3C). Nearly all of the shell-cytochrome samples (except **H**_5_, **R**_5_) exhibited some fraction of the protein sample with properties consistent with much larger radii (>10 nm); these may be attributed to higher-order shell protein assembly. Consistent with this interpretation, DLS analysis of the same samples in a buffer that disrupts hexamer-hexamer interactions (i.e., with 4 M urea) favored a single sharp peak of particle radii at 3–4 nm across all shell-cytochromes (Supplementary Figure 6C). Taken together, DLS measurements agreed with the PAGE results that shell-cytochromes retained the ability to form hexamers. The presence of large particles in DLS and slow-migrating bands in native PAGE also suggested there were higher-order assemblies that were preferentially disrupted by the addition of 4 M urea. However, these analyses by themselves could not indicate if the same types of higher-order assemblies (i.e., nanotubes) were formed by isolated shell-cytochromes.

### Higher-Order Structure Assembly of Shell-Cytochromes

**Figure 4:**
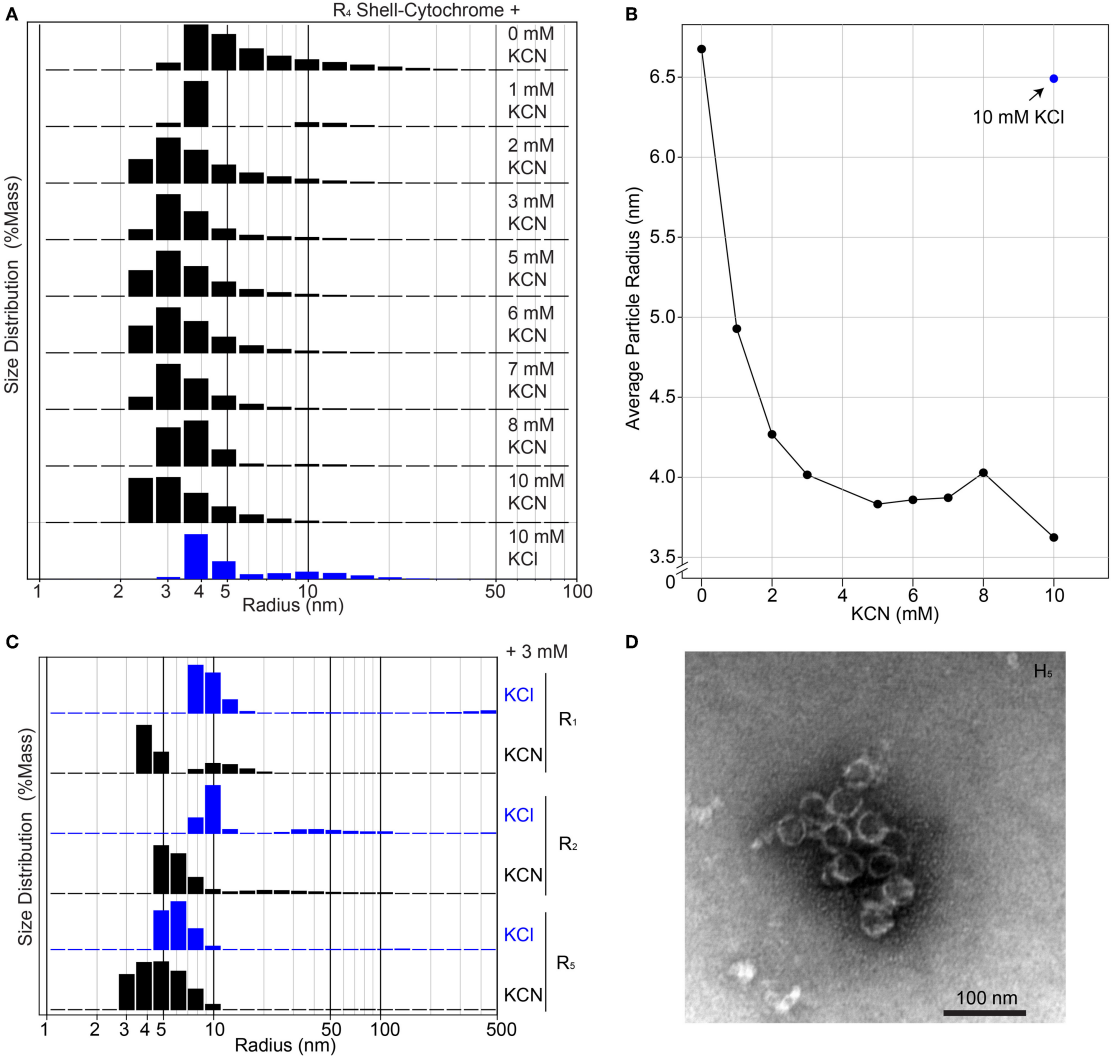
Higher-order assembly of shell-cytochromes. **(A)** Titration of potassium cyanide against 200 μM **R**_4_ shell-cytochrome (based on heme concentration) at pH 7.8 monitored by Dynamic Light Scattering. **(B)** Titration curve of **(A)**, showing the mass-weighted average particle sizes in different concentration of KCN. **(C)** Particle sizes of 200 μM shell-cytochromes **R**_1_, **R**_2_ and **R**_5_ with 3 mM KCN. **(D)** Representative TEM micrograph of spherical shells (often broken) formed by combining **H**_5_ with *H. ochraceum* BMC-P and BMC-T in an *in vitro* assembly reaction, as described in Hagen A. R. et al. (2018).

The unmodified RmmH and Y41A Ho-5815 shell proteins readily form nanotubes that can be observed by electron microscopy (Noël et al., 2016) (Supplementary Figure 1), yet we were unable to find a set of buffer conditions that promoted a similar nanotube formation of shell-cytochrome preparations. However, we consistently observed features that are compatible with the formation of assemblies of multiple hexamers, as exemplified by DLS and PAGE analysis (Figure 3, Supplementary Figure 6). The presence of large structures in shell-cytochromes at low protein concentration suggests that the hemes may mediate unwanted high-affinity protein-protein interactions. As suggested by prior studies of Microperoxidase, such interactions may be driven by intermolecular coordination between iron in hemes and nitrogen atoms within proteins (e.g., 6x His-tag) and/or non-covalent π-interactions between two exposed hemes (Lombardi et al., 2001). To block potential interprotein coordination of hemes, we titrated potassium cyanide (KCN) into purified shell-cytochromes and re-analyzed them by DLS (Figure 4). The amount of **R**_4_ particles that were measured as larger than a single hexamer was inversely proportional to the concentration of KCN (Figures 4A,B). Indeed, **R**_4_ shell-cytochrome exhibited a single peak of particle radii consistent with that of a single hexamer (3.5–4 nm) when an approximately equimolar amount of CN^−^ anions to heme was supplied (Figure 4B). Particle sizes of other shell-cytochromes also decreased when 3 mM KCN was added (Figure 4C). These results were consistent with the hypothesis that inter-protein interactions mediated by hemes may compete with the intrinsic hexamer-hexamer interactions formed by BMC-H proteins, such as a binding between the 6xHis affinity tag and 5-coordinated heme Fe ions.

A second possibility was that shell-cytochrome preparations were unable to form higher-order nanotubes because the proteins are somehow altered in their structure and/or post-translational modifications after being processed through the Sec and heme maturation pathways. As an approach to evaluate this possibility, we constructed another control heme/shell hybrid by appending Ho-5815 with a c-terminal flexible “heme-tag” (**H**_c_) that has been previously reported (Braun et al., 2005). Because the **H**_c_ construct also has a signal peptide, it is processed by the Sec machinery, as are the shell-cytochromes described above. However, in the heme-tag design, a 6xHis-tag is placed immediately adjacent to the heme binding motif (Supplementary Figure 7A), an arrangement that has been shown to favor *intra*-protein coordination of the final axial ligand through the histidine residues of the His-tag contained on the same peptide (Braun et al., 2005). Upon expression and purification of **H**_c_, we found that it was readily capable of forming elongated, nanotube-like bars that could be viewed by EM (Supplementary Figures 7B,C). This suggests that the periplasmic translocation of shell proteins does not alter correct folding, and that heme-bound shell proteins are not inherently unable to form organized higher-order assemblies.

Taken together, the evidence suggests that shell-cytochromes are capable of lateral hexamer-hexamer interactions, but subtle alterations in their self-assembly processes might interfere with native higher-order structure formation. We therefore examined if shell-cytochrome **H**_5_ retained the capability to form the reported compartment assemblies when combined with wild-type *Haliangium ochraceum* (*HO*) BMC shell proteins (Sutter et al., 2017). We performed a recently-described *in vitro* BMC assembly (Hagen A. R. et al., 2018), where three *HO* shell proteins (BMC-H, BMC-P [pentamer], and BMC-T [trimer]) are used to assemble a spherical, hollow protein compartment (Sutter et al., 2017). When **H**_5_ shell-cytochrome was substituted for native BMC-H in these assemblies, we found it associated with native BMC-P and BMC-T proteins, although the strength of the association was greatly enhanced by including wild-type BMC-H in the reactions (Supplementary Figure 8A). Assembly reactions containing only **H**_5_ with native BMC-P and BMC-T, contained higher-order structures resembling the size and shape of *HO* compartments (Figure 4D), although these structures appeared less robust than the published compartments using WT BMC-H proteins (Sutter et al., 2017; Hagen A. et al., 2018; Hagen A. R. et al., 2018; Ferlez et al., 2019). Indeed, **H**_5_-based assemblies frequently appeared as compartments with “broken shells” or as clusters of partially-assembled shell fragments (Supplementary Figure 8B). No discernable compartment- or shell-like assemblies were observed in assembly reactions containing BMC-T and BMC-P only.

## Discussion

Naturally-occurring heme proteins exhibit a large range of functionalities within living cells, including oxygen transport, energy conversion, detoxification (Chapman et al., 1997) and signaling (Liu et al., 1996). In recent years, proteins that bind heme or heme derivatives have become bioengineering targets for conferring novel functions in biological and biohybrid systems, including biosensing (Beissenhirtz et al., 2004), biomemory (Lee et al., 2010; Güzel et al., 2018), biofuel cells (Katz et al., 1999; Ramanavicius and Ramanaviciene, 2009), catalysts (Kleingardner et al., 2014; Firpo et al., 2018) and artificial photosynthesis (Koshiyama et al., 2011; Anderson et al., 2014; Kubie et al., 2017). Therefore, a platform that enables design and synthesis of versatile heme-binding proteins could benefit both fundamental and applied research. In this work, we demonstrated a simple *in vivo* approach that allows *c*-type hemes to be incorporated to non-heme proteins at defined locations (Figure 1A). Our approach could be used to insert a heme attachment site at any location in a target protein, though our results suggest important design considerations for preserving the structure and higher-order assembly capabilities of that target.

Our approach exploits the substrate “promiscuity” of Ccm-I, which may conjugate heme onto virtually any CxxCH motif it is exposed to (Li et al., 2011). In our proof-of-principle studies targeting BMC shell proteins, four of five unique sites achieved high-occupancy of heme incorporation, suggesting Ccm-I is capable of recognizing heme binding motifs mostly independent of other protein contextual features. The majority of engineered shell proteins were determined to have a heme ligated at the intended location, though a proportion of each preparation (<10% to 30%) was composed of the *apo*-protein without a heme attached. It is possible that these represent inefficient Ccm-I heme insertion, though our purification methodology cannot rule out the possibility that these *apo*-proteins are partially a result of proteins that failed to translocate to the periplasm, and therefore remain inaccessible to Ccm-I activity. In support of the second possibility, in preparations with lower heme incorporation (e.g., **H**_1_, **H**_2_, **R**_5_; Figure 2B), we observed a protein band that did not contain hemes on the native gels that migrated consistently slower relative to the homo-hexamer of *holo*-proteins (Supplementary Figure 6A, cyan arrows). This band-pattern indicates that the *apo*-proteins tended to be homo-*apo*-hexamers instead of hetero-(*apo*/*holo*)-hexamers; the latter of which would be expected to possess a binomial distribution ranging from six *apo*-proteins to six *holo*-proteins in the hexamer population (and to thereby exhibit multiple bands on the native gel). Therefore, a parsimonious explanation is that the *apo*-proteins we observed were derived from a cytosolic pool that failed to translocate to the periplasm, instead forming homo-(*apo*)-hexamers in the cytosol.

Heme attachment onto a protein surface by this method appears to maintain many structural features of the unmodified “parental” protein, although we also found evidence that the higher-order assembly of shell-cytochromes may be influenced by the presence of the surface-exposed heme. Shell-cytochromes retained the capacity to hexamerize, as indicated by their similar mobility to hexameric WT shells on native PAGE and the particle diameter measurements via DLS (~3–4 nm; Figure 3). However, we did not observe the formation of nanotubes by shell-cytochromes. It is possible that the covalent attachment of heme may change the geometry or conformation of key residues within the BMC-H subunit in a manner that alters the hexamer-hexamer interface; indeed a number of mutagenesis studies have described BMC-H variants with a single amino acid substitution that form altered higher-order structures (Pang et al., 2014; Sinha et al., 2014; Sutter et al., 2016; Young et al., 2017). Yet, our preliminary data (Supplementary Figures 7, 8) suggest the exposed distal face of a conjugated heme may mediate unwanted protein-protein interactions that compete or interfere with the normal lateral interface between BMC-H hexamers (bis-His heme ligands have a comparable binding energy to predicted hexamer-hexamer interactions (Goff and Morgan, 1976; Greber et al., 2019). Therefore, future engineering efforts may be directed toward minimizing unwanted heme-mediated inter-protein interactions, such as by reducing surface histidine residues or intentionally occupying the heme distal face with other *intra*-protein or chemical ligands.

Although modified shell-cytochromes by themselves did not form identifiable structures, in the presence of shell components (BMC-T and BMC-P), **H**_5_ was successfully incorporated into structures resembling the expected hollow sphere (Figure 4D; Sutter et al., 2017; Hagen A. R. et al., 2018), and compartment fragments. Visualization of multiple assemblies by TEM suggested that **H**_5_ tended to co-assemble into shell fragments (Supplementary Figure 8B), while spherical shell compartments (often appearing “broken”; Figure 4D) were more rare. The amount of **H**_5_ that was co-purified with BMC-P also depended on the amount of WT BMC-H added to the co-purification reaction (Supplementary Figure 8A). Altogether, our results suggest that shell-cytochromes may have altered/impaired higher-order assembly capacity, though they may be successfully incorporated into BMC-based architectures that have more robust assembly properties. Therefore, “doping” a small amount of shell-cytochromes into a BMC shell assembly could install redox carriers that would potentially enable electron flow across the protein layer. In this regard, shell-cytochromes may complement recent efforts to install metallo-centers in shell proteins (Aussignargues et al., 2016; Plegaria and Kerfeld, 2018), with the long-range goal of enabling reductant exchange in and out of engineered microcompartments.

BMC shell proteins are a protein family that is increasingly being developed for a range of synthetic biology applications. Native BMCs are known to encapsulate signature enzymes and partially isolate the lumenal environment from the cytosol, which can increase pathway efficacy by creating a specialized environment. In recent years, great progress has been made in engineering BMC proteins to create designer nanoreactors, including luminal targeting of non-native enzyme “cargo” (Parsons et al., 2010; Cameron et al., 2013; Lawrence et al., 2014; Aussignargues et al., 2015; Quin et al., 2016; Lee et al., 2018; Ferlez et al., 2019), and design of shell proteins with useful biochemical or permeability properties (Klein et al., 2009; Thompson et al., 2014, 2015; Aussignargues et al., 2016; Plegaria and Kerfeld, 2018). The capacity to incorporate heme onto BMC shell proteins could compliment recent efforts to install iron-sulfur clusters within the central pore of shell proteins (Aussignargues et al., 2016), especially as the lateral pore-to-pore distance within a typical BMC shells assembly is too far (>5 nm; Sutter et al., 2017) to support electron transfer at biologically-relevant rates (Page et al., 1999). Increasing the density of electron carriers like the hemes described in this work, could enable construction of self-assembling, electrically conductive architectures. The monoheme shell-cytochromes described here should have 6 redox factors per hexamer, with a separation of ~2–4 nm between hemes (see Supplementary Figure 2A), and di-heme shell-cytochromes (i.e., **H**_2+5_, **R**_2+5_, **R**_4+5_) would have twice the heme density (Supplementary Figure 5). To reach an electron carrier density approximately equal to the highly conductive STC cytochrome (Leys et al., 2002), ~4 redox centers per monomer would be required, though this could be achieved through a mix of redox cofactors (e.g., iron-sulfur clusters and hemes).

Over the decades, novel heme binding proteins have been engineered through a few distinct design approaches (Reedy and Gibney, 2004). Earlier attempts relied upon mutagenesis of naturally occurring heme-containing proteins, like myoglobin, peroxidase, and cytochrome *c*. This approach focuses on generating variants from an existing protein scaffold (Isogai et al., 1999) through alteration of the heme environment, and has interrogated the effect of factors such as axial ligands (Shimizu et al., 1988), solvent exposure (Bortolotti et al., 2011), and the hydrogen bonding network (Goodin and McRee, 1993). On the other end of the spectrum, *de novo* electron-conducting proteins have been designed through attempts to capture salient features of natural heme binding sites and isolate them from convoluted protein folding by developing short polypeptides (i.e., typically 8–30 residues). Examples of this approach include miniature metalloporphyrinyl-peptides such as microperoxidases (Verbaro et al., 2009), peptide-sandwiched mesoheme (Benson et al., 1995), and mimochromes (Lombardi et al., 1998), which can covalently bind heme or heme-analogs, but do not necessarily form well-defined tertiary structures. One successful family of *de novo* heme binding peptides with defined tertiary structures is the maquette (Robertson et al., 1994), a four-helix bundle that forms a heme binding pocket and contains metal ligands positioned at the bundle core (Reedy and Gibney, 2004). Redesigning a structurally-defined, redox-inactive protein so that it is capable of binding heme is a relatively underutilized approach, and tends to involve fusing a new protein domain to a terminus of a target protein with a flexible linker.

As the demand for redox active parts in bioengineering and synthetic biology applications increases, the capacity to add heme to functional protein modules creates potential to build sophisticated architectures with redox and catalytic capabilities (Bostick et al., 2018). The capacity to add heme to a rigid location with well-defined structure is a critical consideration when the applications are related to electron transfer (Matyushov, 2013). Since the geometric constrictions of the heme binding motif have systematically studied (Fufezan et al., 2008; Kozak et al., 2018), the approach we describe could be applied to other protein targets. This system is best-suited for the introduction of hemes upon the surface of a protein, which can be very useful in designs requiring high accessibility of the heme moiety, such as electron transfer (Ruzgas et al., 1999) and catalysis (Kleingardner et al., 2014).

## Materials and Methods

### Identification of Possible Heme Attachment Sites for Shell Proteins Based on Secondary Structures of CxxCH Motif in Natural Cytochrome *c*

Supplementary Table 1 lists 180 cytochrome Protein Data Bank (PDB) entries used for calculating the torsion angles of 249 CxxCH motifs. Based on the plotting of Ramchandra figure, the following gating parameters were used for searching similar secondary structures in Ho-5815 shell protein (the heme binding motif is denoted as x_1_C_1_x_2_x_3_C_2_H): −100° < φ_X1_ < −50°, −180° < ψ_X1_ < +180°; −100° < φ_C1_ < −50°, −100° < ψ_C1_ < +0°; −100° < φ_X2_ < −25°, −75° < ψ_X2_ < +50°; −160° < φ_X3_ < −50°, −75° < ψ_X3_ < +0°; −160° < φ_C2_ < −50°, −75° < ψ_C2_ < +25° and −180° to +180° (no rating) for the last Histidine. Potential regions for heme attachment on Ho-5815 are residues numbers 11–20, 48–60, and 81–82. Further visual checking was performed based on crystal structures to avoid heme from clashing with proteins.

### Strains and Protein Purification

The gene sequences for Ho-5815 Y41A and MSM_0272 were synthesized *de novo* as gBlocks (IDT Biosciences) and inserted into a pETDuet-1 based vector driven by a tac promoter. CxxCH motifs were introduced into the parental shell proteins sequences by designing primers with mutated codons and performing standard PCR mutagenesis, followed by re-annealing of the purified product via isothermal assembly. The coding sequences, including signal peptides and the matured amino acid sequences can be found in Supplementary Table 2.

Heme-attached shell proteins were expressed in *E. coli* BL21 (DE3) (New England Biolabs, MA, USA) strain harboring pEC86 and Heme-attached shell proteins plasmids.

Fresh transformants were first inoculated in 1 mL LB medium overnight, and these starter cultures were then used to inoculate 1 L 2xYT medium (Alpha Biosciences, MD, USA). Cultures were grown at 37°C with continuous shaking (rpm = 140) in an Infors Multitron (Infors Gmbh, Switzerland) for ~6 h to an OD_600_ = 0.8. At this point, shell-cytochrome expression was induced through the addition of 10 μg/mL of 5-aminolevulinic acid (Frontier Scientific, Inc., Utah, USA) and 1 mM Isopropyl β-D-1-thiogalactopyranoside (IPTG; GoldBio, Missouri, USA). Induced cultures were grown at 30°C and 80 rpm for 12 h. Cells were allowed to settle down overnight before discarding the supernatant and harvesting cells. 50 μg/mL Kanamycin (Sigma-Aldrich, MO, USA) and 40 μg/mL Chloramphenicol (Sigma-Aldrich) were added the growth media, as appropriate.

Cells were lysed by sonication (80% Energy 15 min, Model 120 Sonic Dismembrator, Fisher Scientific, Massachusetts, USA) in 50 mM PBS buffer pH 7.0 and the soluble fraction was removed (except **H**_5_, which was soluble and loaded onto resin directly). Red pellets were then resuspended in buffer containing 8 M urea 50 mM Tris and 10 mM imidazole at pH 8.0 and then loaded on 10 mL Ni-NTA super-flow resin (Qiagen, Hilden, Germany) equilibrated with the same buffer. Samples were washed with buffer containing 4 M urea, 50 mM Tris, 500 mM NaCl and 50 mM imidazole at pH 8, and then eluted with buffer containing 4 M urea, 250 mM imidazole, 25 mM Tris, pH 8.0. Purified proteins were dialyzed against buffer containing 50 mM Tris, 100 mM Imidazole, 150 mM NaCl, pH 8.0, then concentrated with Gel-Absorbent (Spectrum Laboratories, Inc., California, USA), and finally dialyzed against buffer containing 50 mM Tris, 100 mM Imidazole, 150 mM NaCl, 10% Glycerol; pH 8.0. Concentrated samples were flash frozen in liquid nitrogen and stored at −80°C for later analysis.

### Molecular Weight Determination by Mass Spectrometry

Purified shell-cytochromes were diluted to ~5 μM (based on the extinction coefficient from the Soret band absorbance) in NH_4_Ac. Five microliters of sample was injected onto a Hypersil Gold cyanopropyl guard column (1 × 10 mm) (Thermo Scientific, Massachusetts, USA) using a Waters Acquity UHPLC system (Waters, Massachusetts, USA). A binary gradient flowing at 0.1 ml min^−1^ to elute the protein was programmed as follows: initial conditions 98% A (water + 0.15% formic acid) / 2% B (acetonitrile), held for 5 min to wash away salts and buffer; a linear ramp to 75% B over 5 min (from 5 to 10 min after loading); hold at 75% B for 2 min (until 12 min after load); return to starting conditions of 98% A / 2% B at 12.01 min and hold 3 min (until 15 min after load) to re-equilibrate the column for the next injection. Proteins were analyzed on a Waters G2-XS quadrupole-time-of-flight mass spectrometer using electrospray ionization operating in positive ion mode and scanning a mass range of m/z 200–2,000 with 1 scan per second. Capillary voltage was 3 kV, sample cone voltage was 35 V, source temperature was 100°C, desolvation temperature was 350°C and desolvation gas flow was 600 L hr^−1^. Elution Peaks at 7–9 min were integrated and the molecular weight spectra were deconvoluted using the MaxEnt 1 algorithm in the Waters Masslynx software package.

### Transmission Electron Microscopy

Thin sections of *E. coli* overexpressing Ho-5815 Y41A were prepared as described (Young et al., 2017). Negative stain of **H**_c_ higher-order protein assembly were prepared following protein purification. Briefly, a ~5 μL droplet was floated on 400-mesh copper carbon only grids (Ted Pella, California, USA) for 5 min, then 1% uranyl acetate was applied to stain the sample for another 5 min. Finally, Reynolds lead citrate was applied for 5 min, the sample was then washed with ultrapure water and dried by wicking with a filter paper. For purified *in vitro* assembly reactions, 5 μL was spotted on 150 mesh carbon coated grids (CF150-CU, Electron Microscopy Sciences, Pennsylvania, USA) for 30 s. Grids were then wicked dry and stained with 5 μL of a 1% uranyl acetate solution for 15 s before again wicking dry and imaging. Thin section images were imaged on a JEM 100CX II (JEOL, Tokyo, Japan) equipped with a Prius SC200-830 CCD camera (Gatan, California, USA), purified protein samples were imaged on JEM-1400Flash (JEOL) equipped with a “Matataki Flash” sCMOS camera (JEOL).

### Molecular Dynamic of Heme-Attached Shell Proteins

Mutations at Sites 1,2,3, and 5 were built by the Mutagenesis Plugin in PyMol (github.com/schrodinger/pymol-open-source). Loop extensions at site 4 were produced by SWISS-MODEL Homology Modeling webserver (Waterhouse et al., 2018). Hemes were loaded to the close proximate to the heme binding motif in PyMol, and then bonded to the cysteines and histidine in VMD (Humphrey et al., 1996) using a modified topology/parameter file (Available in Supplementary Files) based on CHARMM36 (Huang and MacKerell, 2013) toppar_all36_prot_heme.str. The stereochemistry of the Carbon-Sulfur bonds was manually added. All solvated models were minimized for 5,000 steps and the molecular dynamic trajectory at 310 Kelvin for 1 nanosecond were calculated by NAMD (Phillips et al., 2005) (version 2.13 with CUDA) on a lab PC. The snapshot at 1 nanosecond was showed in Figure 1D, and the last 0.8 nanoseconds were used to calculation RMSD in Supplementary Figure 2B.

### UV-Vis Spectroscopy

Frozen Samples were diluted to Absorbance at 408 nm to 0.7–0.8 (1 cm) in a buffer containing 50 mM Tris, 150 mM NaCl, and 100 mM imidazole; pH 8.0. Oxidized samples were measured as prepared from the protein purification described above. To reduce hemes, several crystals of sodium dithionite were added to the sample cuvettes. All spectra were recorded by DU800 (Beckman Coulter, CA, USA). Spectra in Figure 2 were normalized to A_Soret_ = 1.

### Redox Titration

A cocktail of redox mediators (2 μM methyl viologen, 2 μM benzyl viologen, 2 μM Neutral red, 10 μM anthraquinone-2-sulfonate, 10 μM anthraquinone-2,5-disulfonate, 10 μM 2-hydroxy-1,4-naphthaquinone, 10 μM 2,5-dihydroxy-*p*-benzoquinone, 15 μM Pyocyanin, 10 μM 5-hydroxy-1,4-naphthaquinone, and 10 μM potassium ferrocyanide) was added to degassed buffer containing 50 mM Tris 100 mM imidazole pH 8.0. Glassy carbon working electrode and AgCl/Cl reference electrode were used. The reference electrode was calibrated with saturated quinhydrone at pH 7 (${\text{E}}_{\text{m}}^{\prime }$_(AgCl/Cl)_ = +190 mV). Reductant (sodium dithionite, 5 mM or 50 mM) or oxidant (potassium ferricyanide, 5 mM or 50 mM) were titrated into 5 mL of sample that stirred and protected by N_2_. UV-Vis spectra were recorded approximately 2 min after each titration (or until the redox potential reading stabilized within 2 mV) by spectrophotometer (LAMBDA 650, PerkinElmer, Waltham, MA).

### Electrophoresis

Native polyacrylamide gels were cast with 4% acrylamide/bis-acrylamide, pH 8.3 as the stacking gel and 12% Acrylamide/Bis-acrylamide, pH 8.8 as the resolving gel. pH 8.3 was used in stacking gel because the isoelectric point of samples is close to pH 6.8, which as commonly used in PAGE protocols. Frozen samples of the purified shell cytochromes were concentrated within a 30 kDa Amicon 0.5 mL spin column and then diluted to Absorbance at 408 nm to 10 (1 cm). Seven microliters of samples were loaded on the gel. After electrophoresis, gels were first imaged before staining in order to visualize the heme-containing bands separately from bulk protein.

### Dynamic Light Scattering

The same dilution protocol used for Electrophoresis (above) was used to prepare shell-cytochromes for DLS. For analysis under native conditions, samples were used as described without further changes. For analysis of shell-cytochrome light scattering under conditions disfavoring higher-order assembly (4 M Urea), a 1:1 mixture of sample with 8 M Urea 50 mM Tris-Cl pH 8.0 was prepared and measured. All samples were measured in DynaPro NanoStar (Wyatt Technology, California, USA) with 5 s exposure and 25 sample averaging.

### Potassium Cyanide Titration

Shell cytochromes were concentrated using a 50 kDa cutoff Amicon ultrafiltration spin column, and diluted in 50 mM Tris, 100 mM NaCl, containing 0 to 10 mM KCN pH 7.8 (as indicated) to a final absorption of 408 nm approximate to 20 (cm^−1^). DLS were measured with 2 s exposure and 50 sample averaging. The mass-weighted averaged radii of particles were calculated using the distribution of sizes (radius) between 1 and 100 nm. Potassium chloride addition was used as a negative control. The approximate heme concentrations were 200 μM (ε_408nm_ = 100 × 10^3^ cm^−1^). The approximate concentration ionized cyanide anion (CN^−^) was 120 μM for 3 mM KCN at pH 7.8 (pK*a*_HCN_ = 9.21).

### Shell-Cytochrome *in vitro* Assembly and Isolation

The *in vitro* shell self-assembly was performed based on (Hagen A. R. et al., 2018), and is summarized below. SUMO-BMC-H, SUMO-BMC-T and BMC-P proteins were purified as previously described (Hagen A. R. et al., 2018) and these proteins were combined with the **H**_5_ shell-cytochrome from this work (which is a derivative of the Y41A mutant of Ho-5815 BMC-H). Final concentration of 1.25 mg/mL of **H**_5_ was added to a reaction mix containing SUMO-BMC-H, SUMO-BMC-T, BMC-P (Strep-II tagged), and ULP (Maltose binding protein tagged SUMO Protease that removes SUMO from BMC proteins) in buffer containing 50 mM Tris-Cl and 150 mM NaCl at pH 8.0 with a final volume of 200 μL. This assembly mix was incubated at 30°C overnight with no agitation. Assembled shells were enriched by passing the reactions through a Strep-Tactin XT Spin Column (IBA GmbH, Germany), washed four times with 100 μL of buffer containing 50 mM Tris-Cl, 150 mM NaCl pH 8, and eluted with 50 μL buffer containing 100 mM Tris-Cl, 150 mM NaCl, and 50 mM biotin pH 8.0. This procedure allows Strep-II tagged BMC-P to bind to Strep-Tactin, enriching for BMC shells that are bound to the pentamer, including large shell assemblies. Co-purification was analyzed using the same *in vitro* assembly reactions as above. Following overnight incubation, the shell assembly reaction was mixed with 100 μL Strep-Tactin Superflow resin (IBA GmbH, Germany) and incubated at room temperature for 1 h to allow the resin to bind BMC-P and associated shell proteins. Resins was pelleted at 500 x g, washed two times with 1 mL buffer containing 50 mM Tris-Cl and 150 mM NaCl at pH 8.0, then two times with 1 mL buffer containing 50 mM Tris-Cl, 100 mM NaCl, and 150 mM imidazole pH 8.0. Resins was captured on 0.45 μm spin filters and the bound proteins were eluted with 50 μL of buffer containing 50 mM Tris-Cl 150 mM NaCl and 20 mM biotin. The last washing buffer was collected for analysis as was the elution wash containing proteins bound to the resins. Both the last washing buffer and eluted shell proteins were analyzed on SDS-PAGE and visualized by heme-staining.

## Data Availability Statement

The raw data supporting the conclusions of this article will be made available by the authors, without undue reservation, to any qualified researcher.

## Author Contributions

JH conceived project ideas, conducted experiments, analyzed data, and wrote the manuscript. EY and BF conducted experiments, analyzed data, and edited the manuscript. CK, DK, and DD conceived project ideas and wrote and edited the manuscript.

### Conflict of Interest

The authors declare that the research was conducted in the absence of any commercial or financial relationships that could be construed as a potential conflict of interest.
