# Supplementary material for: Functionalization of Bacterial Microcompartment Shell Proteins With Covalently Attached Heme
Source: Front Bioeng Biotechnol. 2020 Jan 9;7:432. doi: 10.3389/fbioe.2019.00432 (PMC6962350; doi:10.3389/fbioe.2019.00432)
Supplement: Supplementary file 4 [file Image_1.pdf]

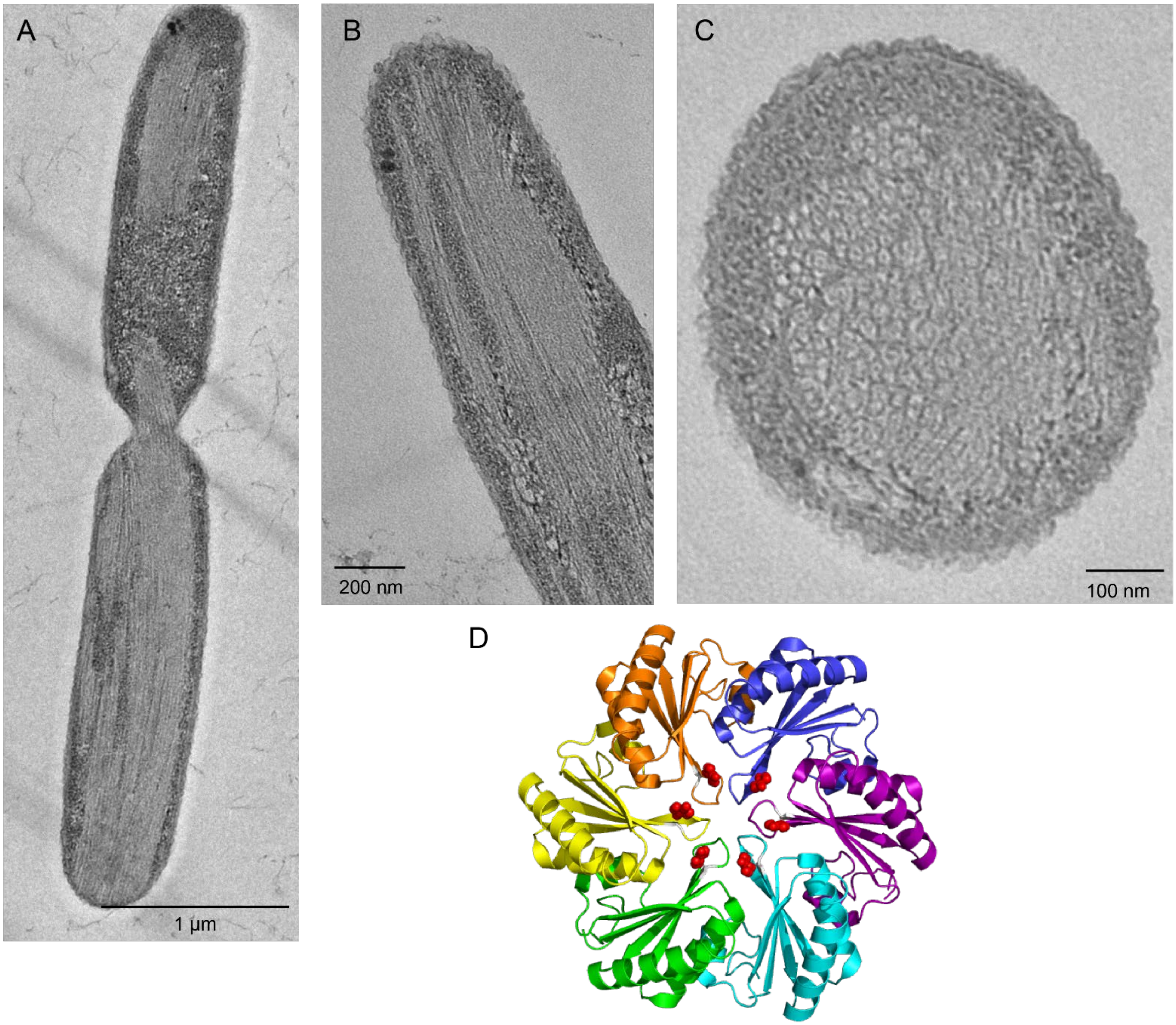

Supplementary Figure 1. Nanotubes formed by Y41A mutant of Ho-5815 during cytosolic overexpression. **(A-C)** Three representative transmission electron microscopy of thin section images of *E. coli* are shown. **(D)** The Y41 of WT Ho-5815 is highlight with red sphere-representation in a hexametric structure model (pdb: 5djb).

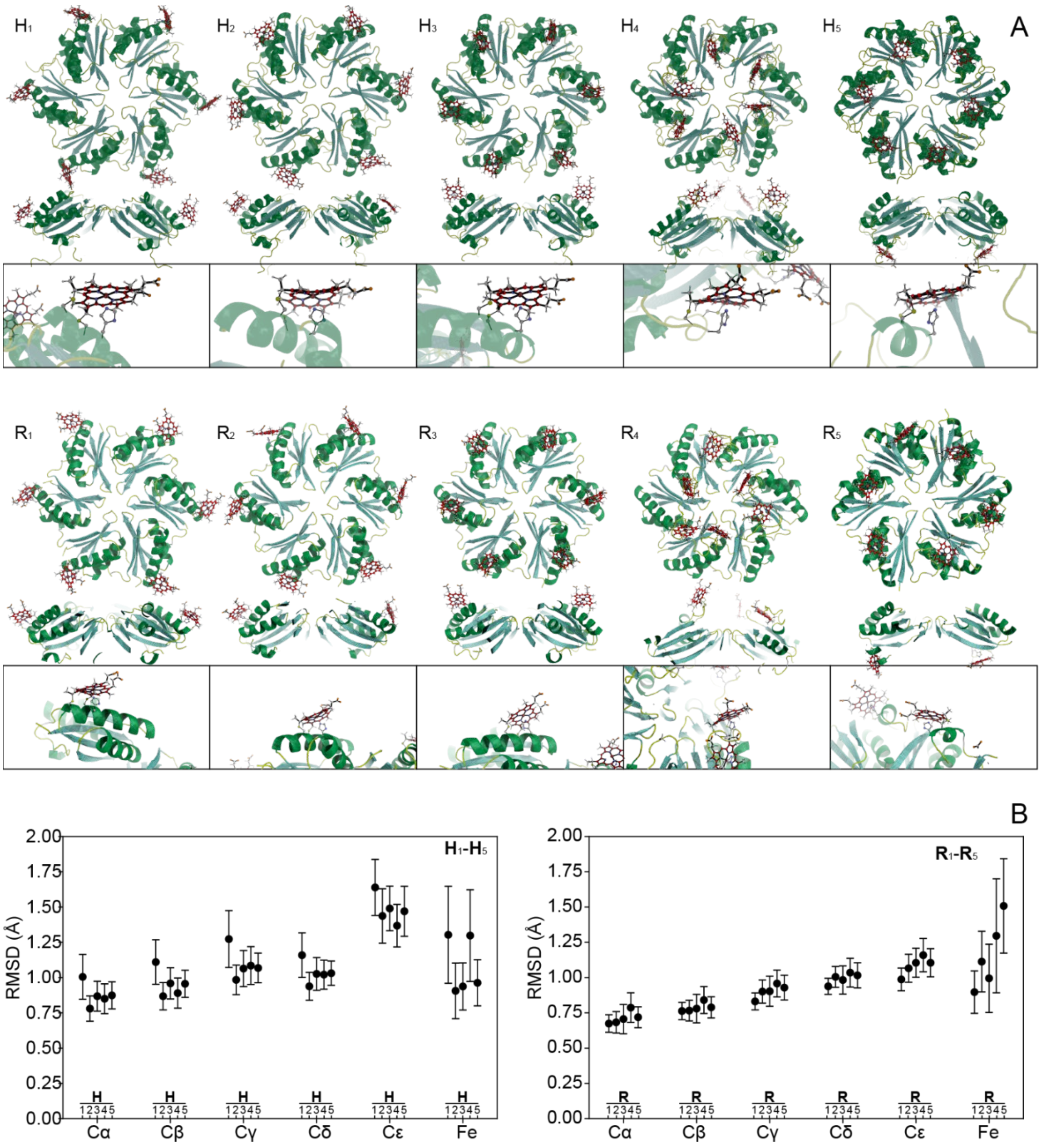

Supplementary Figure 2. Molecular dynamic models of all monoheme shell-cytochromes. **(A)** Top-view, side-view, cross-section, and zoom-in view (Top to bottom) of the predicted structure for each hexameric shell-cytochrome. In the Top-view, Mutation sites 1-4 have the convex side facing out, Mutation site 5 have the concave side facing out. In the Side-view, convex site is oriented upwards. **(B)** Root-mean-square deviation (RMSD) of atomic positions over 0.8 nanoseconds simulation for hexameric shell-cytochromes. C α, Cβ, Cγ, Cδ and Cε are the averaged RMSD of α-carbon to ε-carbon (if exist) of each residue. Fe is the averaged RMSD of iron in six porphyrins for each hexameric shell-cytochrome.

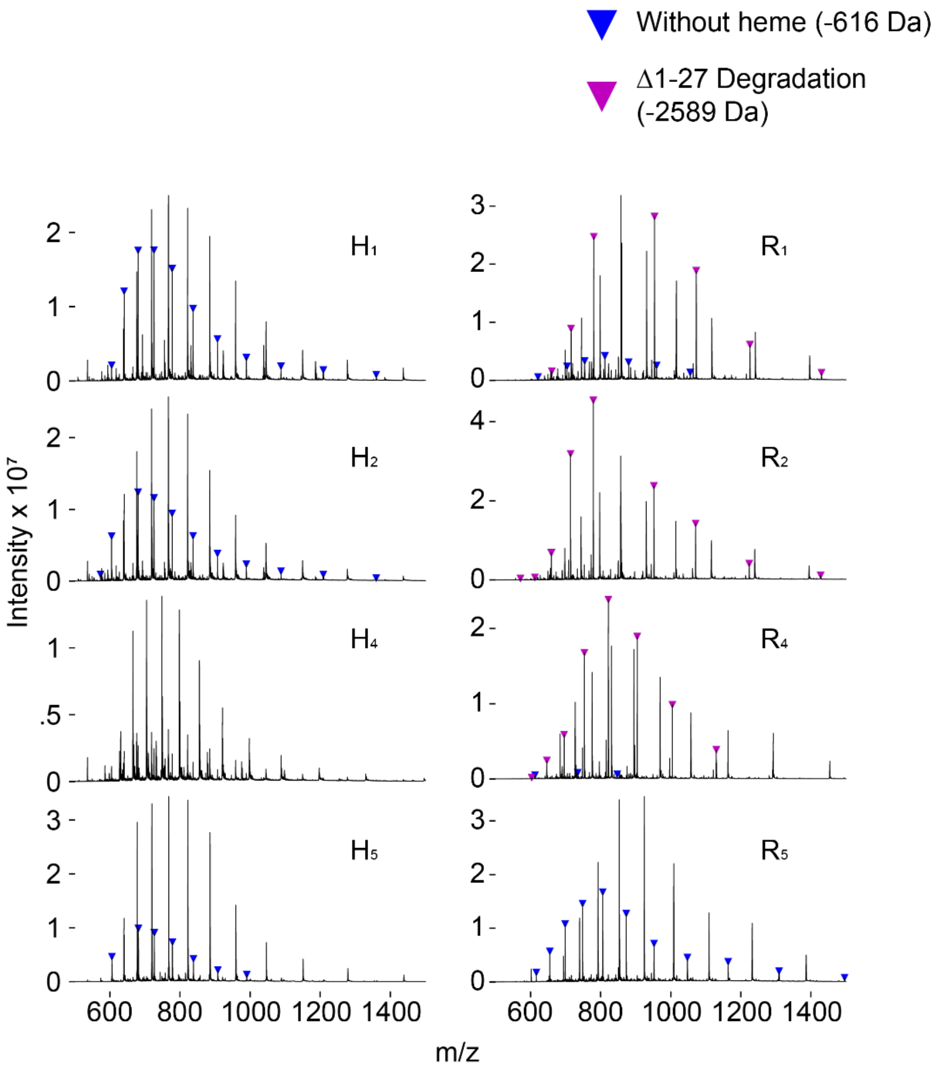

Supplemental Figure 3. Protein mass spectrometry of purified shell-cytochromes. Showing the same results as Figure 2B but marked *apo*-protein (blue triangles). Additionally, a few shell-cytochromes designed using RmmH as the base BMC-H exhibited degradation products lacking the first 27 amino acids on the N-terminus of the protein (magenta triangles).

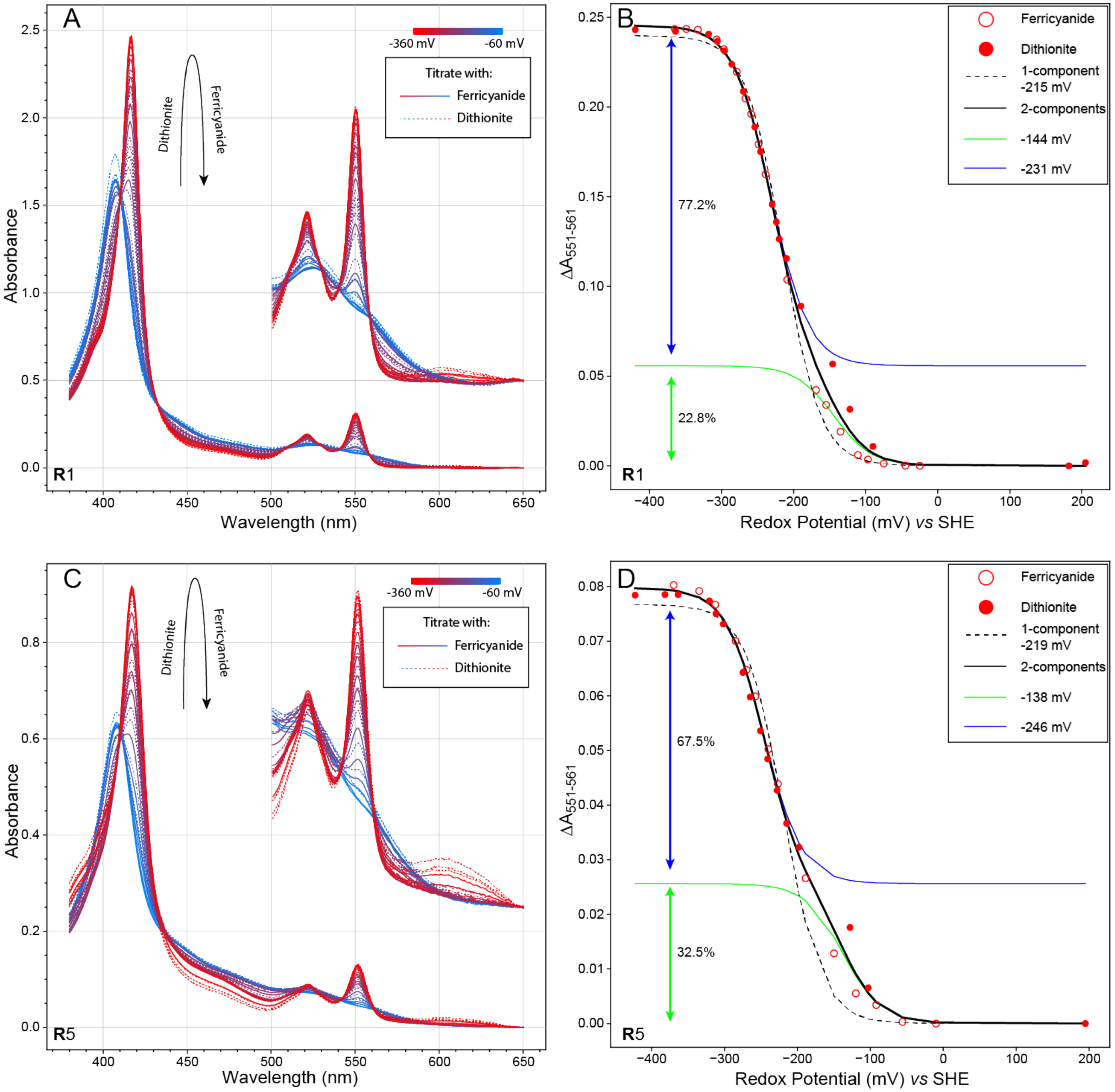

Supplementary Figure 4. Equilibrium redox titration of representative shell-cytochromes, monitored by UV-Vis spectrophotometry. (**A**/**C**) UV-Vis absorption spectra of $\mathbf{R}_1$ and $\mathbf{R}_5$ at different redox potentials (*vs* SHE). Shell-cytochromes were first titrated with sodium dithionite to completely reduce attached heme (dashed traces), then potassium ferricyanide to oxidize the sample (solid traces). The absorbance at wavelengths between 500 – 650 nm is magnified to highlight these spectral features (5x; inset). (**B**/**D**) Titration curves of $\mathbf{R}_1$ (top) and $\mathbf{R}_5$ (bottom). The signal of reduced heme was calculated by the α band (A_{551} – A_{561}, with the minimal absorption normalized to zero). Both potassium ferricyanide (O) and sodium dithionite (●) titration results were fit with the Nernst equation assuming either a single component (black dashed line) or two components (black solid line). Each component of the two-components fitting was plotted in blue (low

potential) or green (high potential) with the estimated ratio of each single component displayed. The two-component E_0' for **R**$_1$ are -144 ± 12 mV (23%) and -231 ± 4 mV (77%); for **R**$_5$, 138 ± 7 mV (32%) and -246 ± 4 mV (68%).

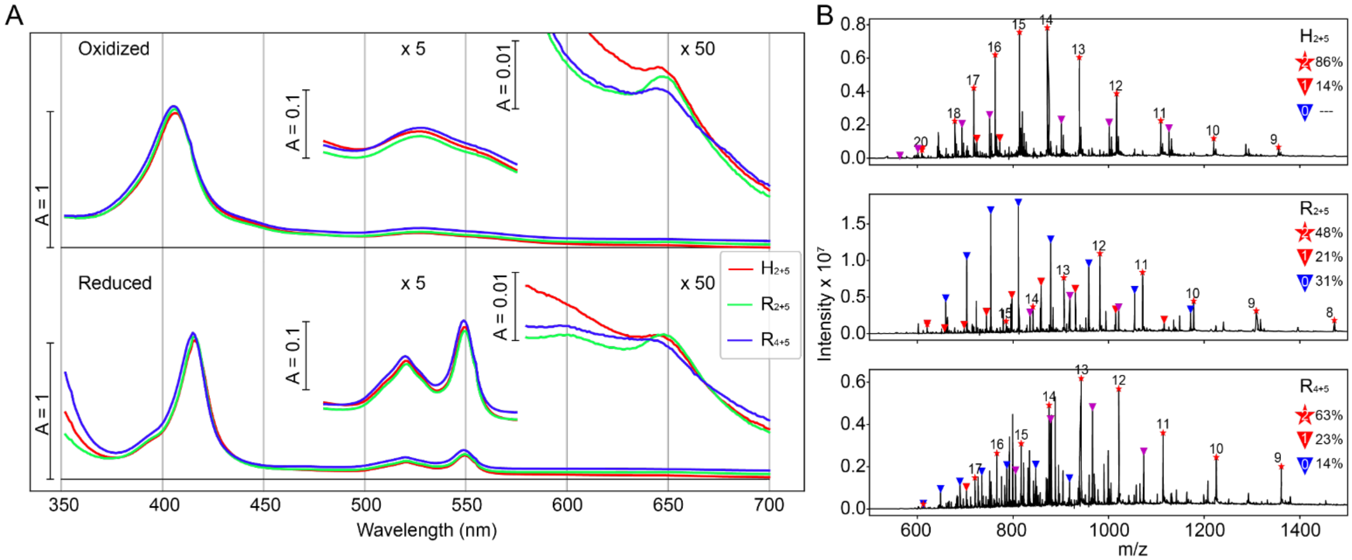

Supplementary Figure 5. Features of purified shell-cytochrome proteins containing two heme attachment sites. (**A)** UV-Vis spectra of the indicated shell-cytochromes under oxidizing (upper) or reducing (lower) conditions. Insets correspond to magnified views of the wavelengths between 480-520 nm (5X magnification) and 580-700 nm (50X). **(B)** Protein mass spectrometry of purified shell-cytochromes **H**$_{2+5}$ (top), **R**$_{2+5}$ (middle), and **R**$_{4+5}$ (bottom). Expected holo-protein are marked with charge states in red stars. The calculated percentage diheme protein (red star), monoheme protein (red triangle) and *apo*-protein (blue triangle) detected in all full-length proteins of each sample are displayed. Truncated proteins are labelled with magenta triangles.

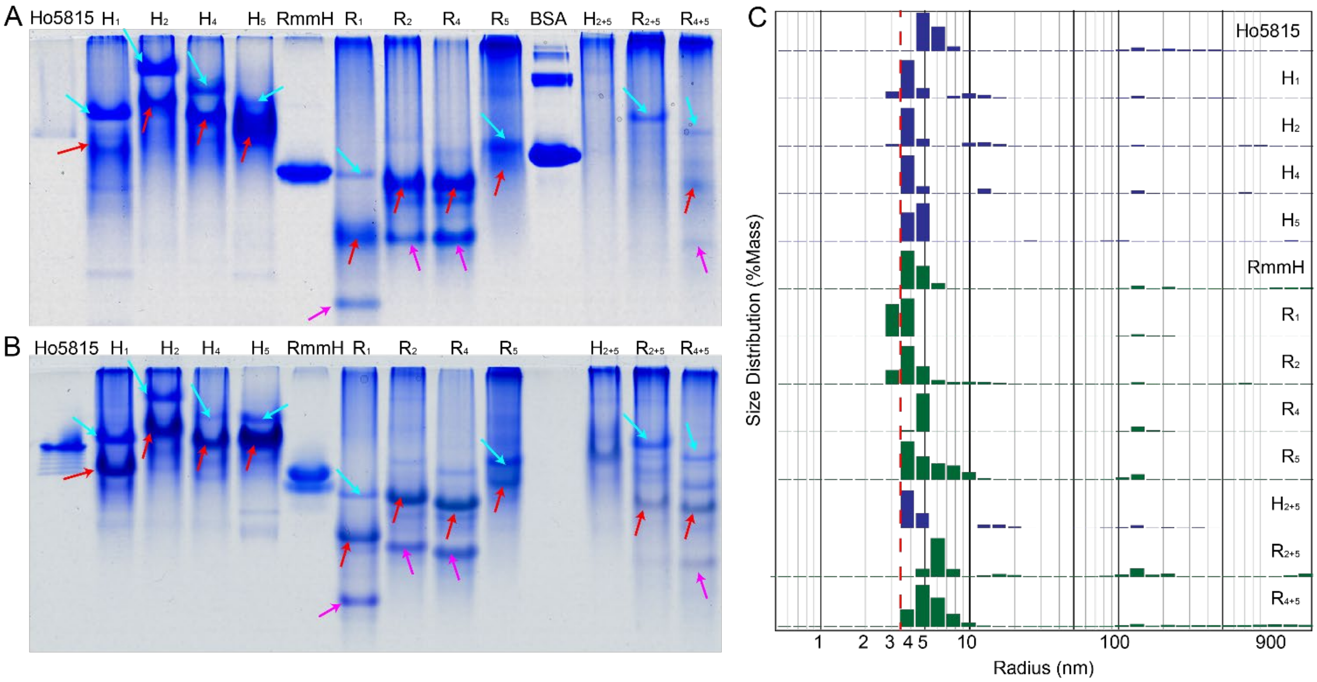

Supplementary Figure 6. Shell-cytochromes retain capacity to oligomerize into hexamers and higher-order assemblies. **(A)** Native PAGE **(B)** native PAGE with 4 M urea show the mobility of **H**-/**R**-variants and their corresponding wild-types. Red arrows point at expected full-length *holo*-shell-cytochromes. Pink arrows point at degraded *holo*-shell-cytochromes detected within the RmmH-derived samples (see Supplemental Figure 3). Cyan arrows point at putative full-length *apo*-shell-cytochromes. **(C)** DLS of **H**-/**R**-variants in the presence of 4 M urea compared against corresponding WT BMC-H proteins. The heights of bars represent the relative mass distribution. The expected 3.5 nm radii for single hexamer shown as red dash-line.

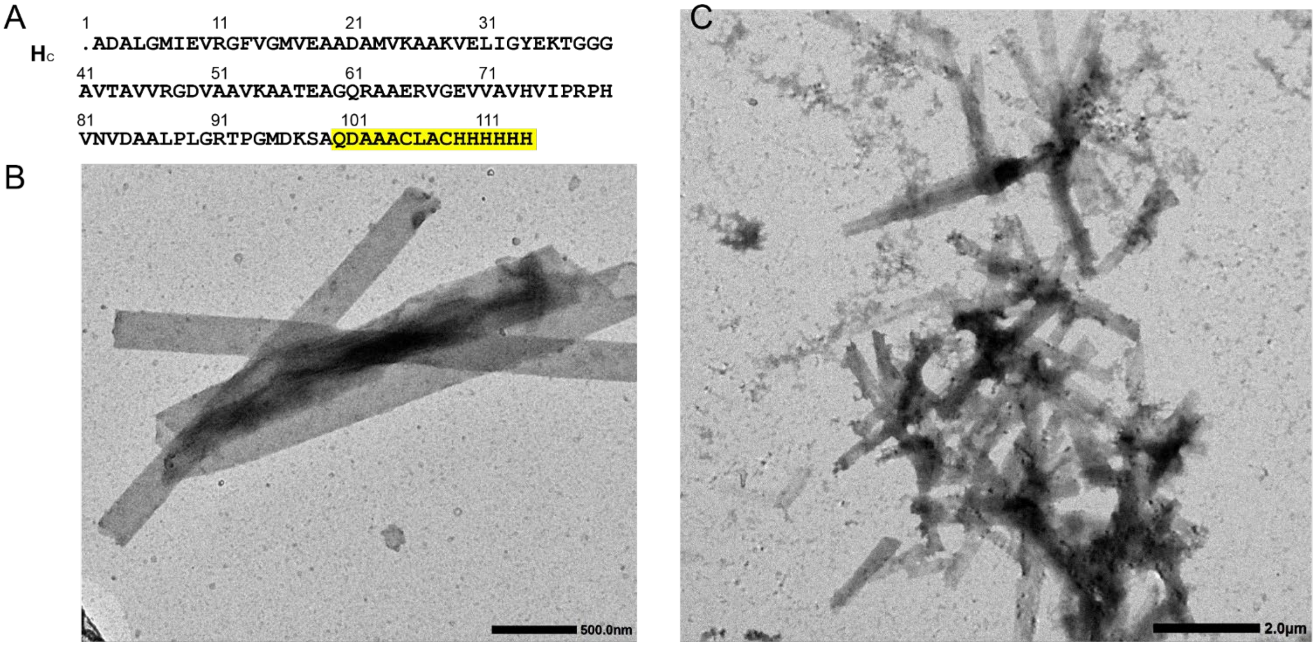

Supplementary Figure 7. Ho-5815 with Y41A mutation and heme-tag at C-terminus (**H**C). **(A)** Sequence of matured **H**C. Residue number 1 is the methionine in WT. In **H**C, a signal peptide was added to the N-terminus, but this signal peptide was removed by peptidase and leaving alanine as the first residue in matured protein. The heme-tag is highlighted. **(B)** & **(C)** transmission electron microscopy of purified **H**C showing the formation of nanotubes.

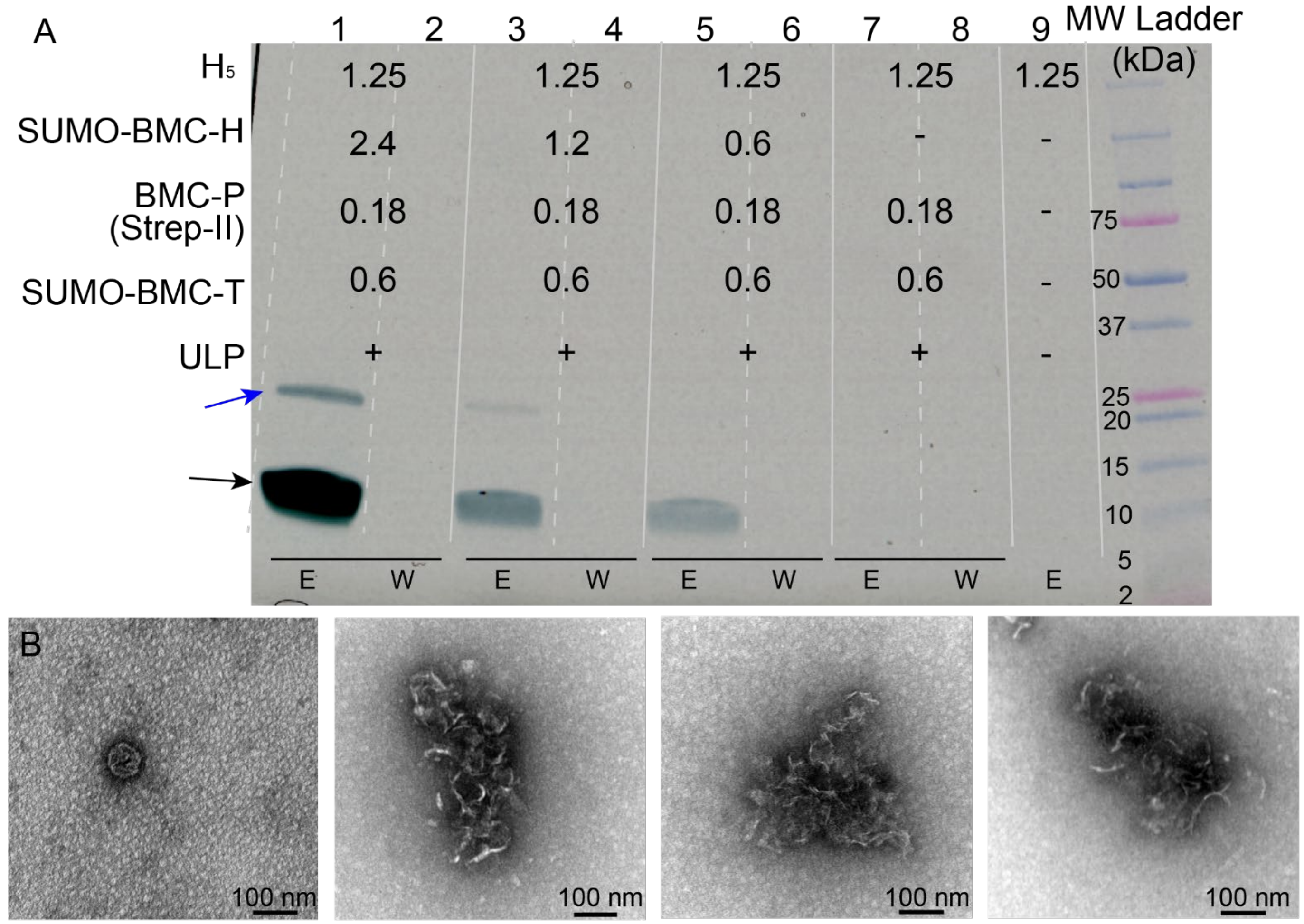

Supplementary Figure 8. Shell protein-protein interactions between shell-cytochrome $\mathbf{H_5}$ and other shell proteins during *in vitro* assemblies of HO shell compartments (see Hagen *et al.*, 2018). **(A)** $\mathbf{H_5}$ associates with BMC-T and BMC-P, as detected in eluates from resin targeting the Strep-II tag encoded on BMC-P. The concentration of each protein (in mg/mL) included in the assembly reaction is reported at the top. Eluted proteins examined by SDS-PAGE and heme staining (staining $\mathbf{H_5}$ shell-cytochrome; black arrow marks expected $\mathbf{H_5}$ monomers, blue arrow marks $\mathbf{H_5}$ dimer). Maltose binding protein tagged SUMO Protease (UDP) was added (+) to cleave the bulky SUMO domains from BMC-H and BMC-T and initiate the assembly reaction. The biotin elutate (E) from the Strep-II resin, and the final supernatant wash (W) from the same reaction were loaded in pairs (*e.g.*, Lanes 1 & 2). **(B)** TEM micrographs from four individual *in vitro* assembly reactions are displayed showing representative images of macromolecular assemblies formed in HO shell *in vitro* assembly containing only $\mathbf{H_5}$ with BMC-P and BMC-T; images include infrequent bodies resembling intact HO shells (left), and large assemblages of resembling "broken shells" (three right images). Scale bars as indicated.
